# Suicide Rates by Industry and Occupation — National Violent Death Reporting System, 32 States, 2016

**DOI:** 10.15585/mmwr.mm6903a1

**Published:** 2020-01-24

**Authors:** Cora Peterson, Aaron Sussell, Jia Li, Pamela K. Schumacher, Kristin Yeoman, Deborah M. Stone

**Affiliations:** ^1^Division of Injury Prevention, National Center for Injury Prevention and Control, CDC; ^2^Spokane Mining Research Division, National Institute for Occupational Safety and Health, CDC; ^3^Division of Field Studies and Engineering, National Institute for Occupational Safety and Health, CDC.

In 2017, nearly 38,000 persons of working age (16–64 years) in the United States died by suicide, which represents a 40% rate increase (12.9 per 100,000 population in 2000 to 18.0 in 2017) in less than 2 decades.* To inform suicide prevention, CDC analyzed suicide data by industry and occupation among working-age decedents presumed to be employed at the time of death from the 32 states participating in the 2016 National Violent Death Reporting System (NVDRS).^†^^,^^§^ Compared with rates in the total study population, suicide rates were significantly higher in five major industry groups: 1) Mining, Quarrying, and Oil and Gas Extraction (males); 2) Construction (males); 3) Other Services (e.g., automotive repair) (males); 4) Agriculture, Forestry, Fishing, and Hunting (males); and 5) Transportation and Warehousing (males and females). Rates were also significantly higher in six major occupational groups: 1) Construction and Extraction (males and females); 2) Installation, Maintenance, and Repair (males); 3) Arts, Design, Entertainment, Sports, and Media (males); 4) Transportation and Material Moving (males and females); 5) Protective Service (females); and 6) Healthcare Support (females). Rates for detailed occupational groups (e.g., Electricians or Carpenters within the Construction and Extraction major group) are presented and provide insight into the differences in suicide rates within major occupational groups. CDC’s Preventing Suicide: A Technical Package of Policy, Programs, and Practices (*1*) contains strategies to prevent suicide and is a resource for communities, including workplace settings.

NVDRS combines data on violent deaths, including suicide, from death certificates, coroner/medical examiner reports, and law enforcement reports. Industry and occupation coding experts used CDC’s National Institute for Occupational Safety and Health Industry and Occupation Computerized Coding System (NIOCCS 3.0)^¶^ to assign 2010 U.S. Census civilian industry and occupation codes for 20,975 suicide decedents aged 16–64 years from the 32 states participating in the 2016 NVDRS, using decedents’ usual industry and occupation as reported on death certificates. Industry (the business activity of a person’s employer or, if self-employed, their own business) and occupation (a person’s job or the type of work they do) are distinct ways to categorize employment (*2*).

Suicide rates were analyzed for industry and occupational groups by sex. Population counts by occupation for rate denominators were states’ civilian, noninstitutionalized current job population counts (for persons aged 16–64 years) from the 2016 American Community Survey Public Use Microdata Sample.** Replicate weight standard errors for those counts were used to calculate 95% confidence intervals (CIs) for suicide rates (*3*). Rates were calculated by U.S. Census code for major industry groups, major occupational groups, and detailed occupational groups with ≥20 decedents; detailed occupational groups are typically more homogenous in terms of employee income, work environment, and peer group. Rates were not calculated for detailed industry groups because many decedents’ industry was classifiable only by major group. The following decedents were excluded from rate calculations: military workers (327); unpaid workers (2,863); those whose other NVDRS data sources (e.g., law enforcement reports) indicated no employment at time of death (i.e., unemployed, disabled, incarcerated, homemaker, or student) (*4*) (1,783); and those not residing in the analysis states (223). A total of 15,779 decedents, including 12,505 (79%) males and 3,274 (21%) females, were included in the analysis. The analysis was conducted using Stata (version 15, StataCorp) and SAS (version 9.4, SAS Institute) statistical software.

Industry and occupational groups with suicide rates significantly (α = 0.05) higher than the study population (i.e., all industries or occupations: 27.4 males [95% CI = 26.9–27.9] and 7.7 females [95% CI = 7.5–8.0] per 100,000 population) were identified when the group’s 95% CI exceeded the study population rate point estimate. Treating the population rate as a constant is reasonable when variance is small and is required for one-sample inference that recognizes the nonindependence of individual industry and occupation groups relative to the study population.

The five major industry groups with suicide rates higher than the study population by sex included 1) Mining, Quarrying, and Oil and Gas Extraction (males: 54.2 per 100,000 civilian noninstitutionalized working population, 95% CI = 44.0–64.3); 2) Construction (males: 45.3, 95% CI = 43.4–47.2); 3) Other Services (e.g., automotive repair; males: 39.1, 95% CI = 36.1–42.0); 4) Agriculture, Forestry, Fishing, and Hunting (males: 36.1, 95% CI = 31.7–40.5); and 5) Transportation and Warehousing (males: 29.8, 95% CI = 27.8–31.9; females: 10.1, 95% CI = 7.9–12.8) (Table 1) (Supplementary Table 1, https://stacks.cdc.gov/view/cdc/84274). The six major occupational groups with higher rates included 1) Construction and Extraction (males: 49.4, 95% CI = 47.2–51.6; females: 25.5, 95% CI = 15.7–39.4); 2) Installation, Maintenance, and Repair (males: 36.9, 95% CI = 34.6–39.3); 3) Arts, Design, Entertainment, Sports, and Media (males: 32.0, 95% CI = 28.2–35.8); 4) Transportation and Material Moving (males: 30.4, 95% CI = 28.8–32.0; females: 12.5, 95% CI = 10.2–14.7); 5) Protective Service (females: 14.0, 95% CI = 9.9–19.2); and 6) Healthcare Support (females: 10.6, 95% CI = 9.2–12.1).

**TABLE 1:** Suicide rates* for persons working in major industry and occupational groups meeting reporting criteria, by sex — National Violent Death Reporting System, 32 states,^†^ 2016^§^

Census code^¶^	Major group	Sex rate (95% CI)	
		Male	Female
**Total**	**All industries or occupations**	**27.4 (26.9–27.9)**	**7.7 (7.5–8.0)**
**Industry**			
0170–0290	Agriculture, Forestry, Fishing, and Hunting	36.1 (31.7–40.5)**	NC^††^
0370–0490	Mining, Quarrying, And Oil and Gas Extraction	54.2 (44.0–64.3)**	NC
0770	Construction	45.3 (43.4–47.2)**	9.4 (6.5–13.2)
1070–3990	Manufacturing	23.6 (22.5–24.8)	7.3 (6.3–8.2)
4070–4590	Wholesale Trade	11.8 (10.1–13.5)	NC
4670–5790	Retail Trade	21.3 (20.0–22.6)	6.8 (6.1–7.5)
6070–6390	Transportation and Warehousing	29.8 (27.8–31.9)**	10.1 (7.9–12.8)**
0570–0690	Utilities	26.3 (21.9–30.7)	NC
6470–6780	Information	19.6 (16.9–22.3)	6.7 (4.7–9.1)
6870–6990	Finance and Insurance	15.1 (13.3–16.8)	6.0 (5.0–6.9)
7070–7190	Real Estate and Rental and Leasing	16.6 (13.8–19.4)	7.1 (5.0–9.7)
7270–7490	Professional, Scientific, and Technical Services	17.6 (16.2–19.0)	6.4 (5.4–7.3)
7570	Management of Companies and Enterprises	NC	NC
7580–7790	Administrative and Support and Waste Management Services	25.9 (23.7–28.1)	5.2 (3.9–6.7)
7860–7890	Educational Services	9.3 (8.1–10.4)	3.9 (3.4–4.4)
7970–8470	Health Care and Social assistance	18.7 (17.0–20.4)	7.5 (7.0–8.0)
8560–8590	Arts, Entertainment, and Recreation	27.4 (24.0–30.8)	9.7 (7.4–12.4)
8660–8690	Accommodation and Food Services	22.9 (21.2–24.6)	7.8 (6.9–8.7)
8770–9290	Other Services	39.1 (36.1–42.0)**	8.8 (7.5–10.0)
9370–9590	Public Administration	23.1 (21.1–25.1)	7.5 (6.2–8.8)
**Occupation**			
0010–0430	Management	17.5 (16.4–18.6)	5.7 (5.0–6.5)
0500–0950	Business and Financial Operations	11.5 (10.0–13.0)	4.7 (3.8–5.5)
1000–1240	Computer and Mathematical	16.2 (14.5–17.9)	6.4 (4.5–8.9)
1300–1560	Architecture and Engineering	23.2 (20.6–25.7)	8.2 (4.7–13.4)
1600–1965	Life, Physical, and Social science	21.4 (16.3–27.6)	5.3 (3.0–8.6)
2000–2060	Community and Social Service	15.4 (11.7–20.0)	6.2 (4.7–8.2)
2100–2160	Legal	16.3 (12.1–21.7)	7.9 (5.4–11.2)
2200–2550	Education, Training, and Library	9.9 (8.3–11.6)	3.9 (3.3–4.6)
2600–2960	Arts, Design, Entertainment, Sports, and Media	32.0 (28.2–35.8)**	8.8 (6.7–11.5)
3000–3540	Healthcare Practitioners and Technical	23.6 (20.8–26.3)	8.5 (7.6–9.4)
3600–3655	Healthcare Support	23.6 (17.0–32.1)	10.6 (9.2–12.1)**
3700–3955	Protective Service	26.4 (23.7–29.1)	14.0 (9.9–19.2)**
4000–4160	Food Preparation and Serving Related	21.1 (19.2–22.9)	7.8 (6.7–8.8)
4200–4250	Building and Grounds Cleaning and Maintenance	26.7 (24.4–29.0)	6.9 (5.3–8.7)
4300–4650	Personal Care and Service	25.0 (21.2–28.8)	8.4 (7.2–9.5)
4700–4965	Sales and Related	20.7 (19.3–22.1)	7.1 (6.3–7.8)
5000–5940	Office and Administrative Support	14.2 (12.9–15.5)	5.4 (4.9–5.9)
6000–6130	Farming, Fishing, and Forestry	31.4 (25.6–37.1)	NC
6200–6940	Construction and Extraction	49.4 (47.2–51.6)**	25.5 (15.7–39.4)**
7000–7630	Installation, Maintenance, and Repair	36.9 (34.6–39.3)**	NC
7700–8965	Production	27.5 (25.9–29.2)	6.8 (5.6–8.1)
9000–9750	Transportation and Material Moving	30.4 (28.8–32.0)**	12.5 (10.2–14.7)**

**Abbreviations**: CI = confidence interval; NC = not calculated.

* Per 100,000 civilian, noninstitutionalized working persons aged 16–64 years.

^†^ Alaska, Arizona, Colorado, Connecticut, Georgia, Hawaii, Illinois, Indiana, Iowa, Kansas, Kentucky, Maine, Maryland, Massachusetts, Michigan, Minnesota, New Hampshire, New Jersey, New Mexico, New York, North Carolina, Ohio, Oklahoma, Oregon, Pennsylvania, Rhode Island, South Carolina, Utah, Vermont, Virginia, Washington, and Wisconsin.

^§^ Number of suicide decedents = 15,779.

^¶^ Census Bureau 2012 industry and 2010 occupational codes from the 2016 American Community Survey, translated from National Institute for Occupational Safety and Health Industry and Occupation Computerized Coding System codes using Census Bureau definitions (https://www.census.gov/topics/employment/industry-occupation/guidance/code-lists.html).

** Statistically higher than population rate (all industries or occupations) based on 95% CI of industry or occupational group rate not containing the total population rate point estimate.

^††^ NC indicates that rate was not calculated because the number of decedents was <20.

Rates could be calculated for 118 detailed occupational groups for males and 32 for females (Supplementary Table 2, https://stacks.cdc.gov/view/cdc/84275). Some occupational groups with suicide rates significantly higher than those of the study population were only identifiable through observation at the detailed group level (Table 2). Among males, these detailed groups included the following seven groups: 1) Fishing and hunting workers (part of the Farming, Fishing, and Forestry major occupational group); 2) Machinists (Production major group); 3) Welding, soldering, and brazing workers (Production major group); 4) Chefs and head cooks (Food Preparation and Serving Related major group); 5) Construction managers (Management major group); 6) Farmers, ranchers, and other agricultural managers (Management major group); and 7) Retail salespersons (Sales and Related major group). Among females, these detailed groups included the following five groups: 1) Artists and related workers (Arts, Design, Entertainment, Sports, and Media major group); 2) Personal care aides (Personal Care and Service major group); 3) Retail salespersons (Sales and Related major group); 4) Waiters and waitresses (Food Preparation and Serving Related major group); and 5) Registered nurses (Healthcare Practitioners and Technical major group). Groups with highest rate point estimates (e.g., female Artists and related workers and male Fishing and hunting workers) also had wide 95% CIs (Table 2), based on relatively low numbers of decedents and relatively small working populations (Supplementary Table 2, https://stacks.cdc.gov/view/cdc/84275).

**TABLE 2:** Detailed occupational groups meeting reporting criteria with male and female suicide rates* higher^†^ than the population rate (all occupations) and associated major occupational groups and rates — National Violent Death Reporting System, 32 states,^§^ 2016^¶^

Sex/Census code**	Detailed occupational group	Rate (95% CI)^†^	Census code**	Part of major occupational group	Rate (95% CI)
**Male**					
6100	Fishing and hunting workers	119.9 (60.9–215.6)^†^	6000–6130	Farming, Fishing, and Forestry	31.4 (25.6–37.1)
2750	Musicians, singers, and related workers	96.5 (63.7–141.1)^†^	2600–2960	Arts, Design, Entertainment, Sports, and Media	32.0 (28.2–35.8)^†^
2600	Artists and related workers	93.5 (60.7–138.5)^†^	2600–2960	Arts, Design, Entertainment, Sports, and Media	32.0 (28.2–35.8)^†^
6530	Structural iron and steel workers	79.0 (43.5–134.0)^†^	6200–6940	Construction and Extraction	49.4 (47.2–51.6)^†^
7360	Millwrights	78.7 (39.8–142.4)^†^	7000–7630	Installation, Maintenance, and Repair	36.9 (34.6–39.3)^†^
6220	Brickmasons, blockmasons, stonemasons, and reinforcing iron and rebar workers	67.6 (45.7–97.0)^†^	6200–6940	Construction and Extraction	49.4 (47.2–51.6)^†^
6515	Roofers	65.2 (46.1–90.0)^†^	6200–6940	Construction and Extraction	49.4 (47.2–51.6)^†^
7200	Automotive service technicians and mechanics	64.8 (57.4–72.3)^†^	7000–7630	Installation, Maintenance, and Repair	36.9 (34.6–39.3)^†^
8030	Machinists	64.2 (53.1–75.3)^†^	7700–8965	Production	27.5 (25.9–29.2)
6260	Construction laborers	62.0 (56.7–67.3)^†^	6200–6940	Construction and Extraction	49.4 (47.2–51.6)^†^
7010	Computer, automated teller, and office machine repairers	60.8 (41.8–86.1)^†^	7000–7630	Installation, Maintenance, and Repair	36.9 (34.6–39.3)^†^
6240	Carpet, floor, and tile installers and finishers	55.2 (35.3–83.1)^†^	6200–6940	Construction and Extraction	49.4 (47.2–51.6)^†^
7150	Automotive body and related repairers	54.9 (34.4–83.9)^†^	7000–7630	Installation, Maintenance, and Repair	36.9 (34.6–39.3)^†^
6230	Carpenters	54.7 (49.0–60.4)^†^	6200–6940	Construction and Extraction	49.4 (47.2–51.6)^†^
8140	Welding, soldering, and brazing workers	53.6 (45.2–62.1)^†^	7700–8965	Production	27.5 (25.9–29.2)
6320	Construction equipment operators except paving, surfacing, and tamping equipment operators	52.8 (42.2–63.4)^†^	6200–6940	Construction and Extraction	49.4 (47.2–51.6)^†^
9620	Laborers and freight, stock, and material movers, hand	51.5 (47.1–55.8)^†^	9000–9750	Transportation and Material Moving	30.4 (28.8–32.0)^†^
4000	Chefs and head cooks	47.8 (38.3–57.2)^†^	4000–4160	Food Preparation and Serving Related	21.1 (19.2–22.9)
0220	Construction managers	45.7 (38.4–53.1)^†^	0010–0430	Management	17.5 (16.4–18.6)
6355	Electricians	44.0 (37.7–50.2)^†^	6200–6940	Construction and Extraction	49.4 (47.2–51.6)^†^
6200	First-line supervisors of construction trades and extraction workers	44.0 (37.4–50.5)^†^	6200–6940	Construction and Extraction	49.4 (47.2–51.6)^†^
0205	Farmers, ranchers, and other agricultural managers	43.2 (34.9–51.5)^†^	0010–0430	Management	17.5 (16.4–18.6)
6420	Painters and paperhangers	36.6 (29.4–43.9)^†^	6200–6940	Construction and Extraction	49.4 (47.2–51.6)^†^
6440	Pipelayers, plumbers, pipefitters, and steamfitters	35.4 (28.7–42.1)^†^	6200–6940	Construction and Extraction	49.4 (47.2–51.6)^†^
4760	Retail salespersons	31.3 (27.7–35.0)^†^	4700–4965	Sales and Related	20.7 (19.3–22.1)
9130	Driver/sales workers and truck drivers	30.4 (27.8–33.0)^†^	9000–9750	Transportation and Material Moving	30.4 (28.8–32.0)^†^
**Total**	**All occupations**	**27.4 (26.9–27.9)**			
**Female**					
2600	Artists and related workers	45.5 (25.7–75.5)^†^	2600–2960	Arts, Design, Entertainment, Sports, and Media	8.8 (6.7–11.5)
9620	Laborers and freight, stock, and material movers, hand	20.9 (14.9–28.8)^†^	9000–9750	Transportation and Material Moving	12.5 (10.2–14.7)^†^
4610	Personal care aides	12.1 (9.0–16.0)^†^	4300–4650	Personal Care and Service	8.4 (7.2–9.5)
4760	Retail salespersons	11.5 (9.3–13.7)^†^	4700–4965	Sales and Related	7.1 (6.3–7.8)
4110	Waiters and waitresses	11.3 (9.1–13.4)^†^	4000–4160	Food Preparation and Serving Related	7.8 (6.7–8.8)
3600	Nursing, psychiatric, and home health aides	10.2 (8.3–12.0)^†^	3600–3655	Healthcare Support	10.6 (9.2–12.1)^†^
3255	Registered nurses	10.1 (8.6–11.6)^†^	3000–3540	Healthcare Practitioners and Technical	8.5 (7.6–9.4)
**Total**	**All occupations**	**7.7 (7.5–8.0)**			

**Abbreviation:** CI = confidence interval.

* Per 100,000 civilian, noninstitutionalized working persons aged 16–64 years.

^†^ Statistically higher than population rate (all occupations) based on 95% CI of occupational group rate not containing the total population rate point estimate.

^§^ Alaska, Arizona, Colorado, Connecticut, Georgia, Hawaii, Illinois, Indiana, Iowa, Kansas, Kentucky, Maine, Maryland, Massachusetts, Michigan, Minnesota, New Hampshire, New Jersey, New Mexico, New York, North Carolina, Ohio, Oklahoma, Oregon, Pennsylvania, Rhode Island, South Carolina, Utah, Vermont, Virginia, Washington, and Wisconsin.

^¶^ Number of suicide decedents = 15,779.

** Census Bureau 2012 industry and 2010 occupational codes from the 2016 American Community Survey, translated from National Institute for Occupational Safety and Health Industry and Occupation Computerized Coding System codes using Census Bureau definitions (https://www.census.gov/topics/employment/industry-occupation/guidance/code-lists.html).

## Discussion

This report used data from 32 states to provide updated population-level suicide rates for major occupational groups and new information on suicide rates for major industry groups and detailed occupational groups. Estimates for most major occupational groups are similar, although not directly comparable, to previous estimates that were based on 2015 NVDRS data from 17 states (*4*). Recent NVDRS expansion to 50 states might facilitate direct comparisons over time by industry and occupation nationwide. These findings highlight opportunities for targeted prevention strategies and further investigation of work-related factors that might increase risk of suicide. Previous research indicates suicide risk is associated with low-skilled work (*5*), lower education (*6*), lower absolute and relative socioeconomic status (*7*), work-related access to lethal means (*8*), and job stress, including poor supervisory and colleague support, low job control, and job insecurity (*9*). Industry, labor, and professional associations, as well as employers, and state and local health departments can use this information to focus attention and resources on suicide prevention. Future research might examine these and other risk factors among the industries and occupations identified in this report as having high suicide rates.

This report estimated suicide rates comprehensively for industry and occupational groups meeting sample size criteria and identified groups with rates higher than the study’s population rate. Although relative comparisons of suicide rates in this manner are useful for prevention purposes, these results should not overshadow the essential fact that the suicide rate in the U.S. working-age population overall has increased by 40% in less than 2 decades. Therefore, all industry sectors and occupational groups can contribute to reducing suicide incidence.

The findings in this report are subject to at least five limitations. First, this study did not address confounding factors that might account for different suicide rates among and within industry or occupational groups. Second, it did not address suicide among unemployed decedents, military or unpaid workers, or those aged >64 years (*9*). Third, the numerator and denominator data were not a direct match for calculating rates; death certificates reflect decedents’ usual industry and occupation, and available population size data refer to the number of persons by current job. Fourth, the results are based on data from 32 states and are therefore not nationally representative. Finally, three states contributing to the 2016 NVDRS did not collect data on all violent deaths. Other limitations of NVDRS analysis using death certificate industry and occupation data have been described previously (*4*).

All industries and occupations can benefit from a comprehensive approach to suicide prevention. CDC’s Preventing Suicide: A Technical Package of Policy, Programs, and Practices (*1*) provides strategies with the best available evidence to prevent suicide and can serve as a resource for communities and employers. Workplace-specific strategies include 1) promoting help-seeking; 2) integrating workplace safety and health and wellness programs to advance the overall well-being of workers; 3) referring workers to financial and other helping services; 4) facilitating time off and benefits to cover supportive services; 5) training personnel to detect and appropriately respond to suicide risk; 6) creating opportunities for employee social connectedness; 7) reducing access to lethal means among persons at risk; and 8) creating a crisis response plan sensitive to the needs of coworkers, friends, family, and others who might themselves be at risk (*1*,*10*). Other community-based strategies include strengthening economic supports, strengthening access and delivery of care, teaching coping and problem-solving skills, and responsibly reporting suicide (e.g., not providing details) (*1*). Further workplace prevention resources are available at https://workplacesuicideprevention.com/ and https://theactionalliance.org/communities/workplace and help is available at 1-800-273-TALK (8255).

**Summary**

**What is already known about this topic?**

Suicide among the U.S. working-age population (ages 16–64 years) is increasing; in 2017, nearly 38,000 persons died by suicide.

**What is added by this report?**

National Violent Death Reporting System data from 32 states were used to calculate suicide rates for major industry and occupational groups and detailed occupational groups. Five industry groups and six major occupational groups had higher suicide rates than did the overall study population. Suicide rates for detailed occupational groups provide insight into subcategories within major groups.

**What are the implications for public health practice?**

Opportunities exist for targeted and broadscale prevention. CDC’s Preventing Suicide: A Technical Package of Policy, Programs, and Practices provides strategies to prevent suicide and can serve as a resource for communities and employers.

## Footnotes

* https://www.cdc.gov/injury/wisqars.

† https://www.cdc.gov/violenceprevention/nvdrs.

§ In 2016, 32 states participated in NVDRS: Alaska, Arizona, Colorado, Connecticut, Georgia, Hawaii, Illinois, Indiana, Iowa, Kansas, Kentucky, Maine, Maryland, Massachusetts, Michigan, Minnesota, New Hampshire, New Jersey, New Mexico, New York, North Carolina, Ohio, Oklahoma, Oregon, Pennsylvania, Rhode Island, South Carolina, Utah, Vermont, Virginia, Washington, and Wisconsin. That year, Illinois, Pennsylvania, and Washington each collected data on ≥80% of violent deaths in the state, in accordance with requirements under which the state was funded for NVDRS; therefore, presented data likely underestimate suicide deaths and rates.

¶ https://wwwn.cdc.gov/nioccs3.

** https://www.census.gov/programs-surveys/acs/data/pums.html.
